# A Responsible Framework for Assessing, Selecting, and Explaining Machine Learning Models in Cardiovascular Disease Outcomes Among People With Type 2 Diabetes: Methodology and Validation Study

**DOI:** 10.2196/66200

**Published:** 2025-06-27

**Authors:** Yang Yang, Che-Yi Liao, Esmaeil Keyvanshokooh, Hui Shao, Mary Beth Weber, Francisco J Pasquel, Gian-Gabriel P Garcia

**Affiliations:** 1 H. Milton Stewart School of Industrial and Systems Engineering, Georgia Institute of Technology, 765 Ferst Dr NW, Atlanta, GA, 30332-0001, United States, 1 404-385-3140; 2 Department of Information and Operations Management, Mays Business School, Texas A&M University, College Station, TX, United States; 3 Hubert Department of Global Health, Rollins School of Public Health, Emory University, Atlanta, GA, United States; 4 Division of Endocrinology, Metabolism, and Lipids, Department of Medicine, Emory University School of Medicine, Atlanta, GA, United States

**Keywords:** interpretable machine learning, explainability, fairness, type 2 diabetes, cardiovascular disease, responsible framework, cardiovascular, cardiology, machine learning, diabetes, clinical practice, myocardial infarction, MI, stroke, prediction, T2D

## Abstract

**Background:**

Building machine learning models that are interpretable, explainable, and fair is critical for their trustworthiness in clinical practice. Interpretability, which refers to how easily a human can comprehend the mechanism by which a model makes predictions, is often seen as a primary consideration when adopting a machine learning model in health care. However, interpretability alone does not necessarily guarantee explainability, which offers stakeholders insights into a model’s predicted outputs. Moreover, many existing frameworks for model evaluation focus primarily on maximizing predictive accuracy, overlooking the broader need for interpretability, fairness, and explainability.

**Objective:**

This study proposes a 3-stage machine learning framework for responsible model development through model assessment, selection, and explanation. We demonstrate the application of this framework for predicting cardiovascular disease (CVD) outcomes, specifically myocardial infarction (MI) and stroke, among people with type 2 diabetes (T2D).

**Methods:**

We extracted participant data comprised of people with T2D from the ACCORD (Action to Control Cardiovascular Risk in Diabetes) dataset (N=9635), including demographic, clinical, and biomarker records. Then, we applied hold-out cross-validation to develop several interpretable machine learning models (linear, tree-based, and ensemble) to predict the risks of MI and stroke among patients with diabetes. Our 3-stage framework first assesses these models via predictive accuracy and fairness metrics. Then, in the model selection stage, we quantify the trade-off between accuracy and fairness using area under the curve (AUC) and Relative Parity of Performance Scores (RPPS), wherein RPPS measures the greatest deviation of all subpopulations compared with the population-wide AUC. Finally, we quantify the explainability of the chosen models using methods such as SHAP (Shapley Additive Explanations) and partial dependence plots to investigate the relationship between features and model outputs.

**Results:**

Our proposed framework demonstrates that the GLMnet model offers the best balance between predictive performance and fairness for both MI and stroke. For MI, GLMnet achieves the highest RPPS (0.979 for gender and 0.967 for race), indicating minimal performance disparities, while maintaining a high AUC of 0.705. For stroke, GLMnet has a relatively high AUC of 0.705 and the second-highest RPPS (0.961 for gender and 0.979 for race), suggesting it is effective across both subgroups. Our model explanation method further highlights that the history of CVD and age are the key predictors of MI, while HbA_1c_ and systolic blood pressure significantly influence stroke classification.

**Conclusions:**

This study establishes a responsible framework for assessing, selecting, and explaining machine learning models, emphasizing accuracy-fairness trade-offs in predictive modeling. Key insights include: (1) simple models perform comparably to complex ensembles; (2) models with strong accuracy may harbor substantial differences in accuracy across demographic groups; and (3) explanation methods reveal the relationships between features and risk for MI and stroke. Our results underscore the need for holistic approaches that consider accuracy, fairness, and explainability in interpretable model design and selection, potentially enhancing health care technology adoption.

## Introduction

Building trustworthy machine learning models for clinical practice requires consideration of interpretability, explainability, as well as fairness. Interpretability—which refers to how easily a human can comprehend the mechanism by which a model makes predictions—is important in health care settings because of the need for clinicians and patients to understand and trust the Artificial Intelligence (AI)-involved decisions that directly impact patient care [1,2]. It also facilitates regulatory compliance and ethical considerations in medical AI applications, ensuring these systems are not only effective but also justifiable and accountable [3]. In addressing this pressing need, experts in computer science, operations research, and medical informatics have significantly progressed the field of interpretable machine learning models, laying the foundation for the development of AI [3,4]. As of today, cutting-edge interpretable machine learning models are available through many open-source software packages, including RiskSLIM and Interpretable AI (Interpretable AI), among others [5,6,7]. Nonetheless, interpretability alone is not sufficient for trustworthy AI in health care.

Beyond interpretability, the machine learning community has also begun to emphasize the need for explainability [8] which focuses on conveying understandable reasons behind AI-driven decisions. While interpretability helps users grasp how a model arrives at a conclusion, explainability provides the why, offering justifications in human terms. This reasoning is crucial in health care, where clinicians and patients must not only understand but also trust the rationale of AI suggestions [9]. Therefore, explainability builds trust, enhances decision-making quality by providing insights into AI reasoning, and ensures compliance with ethical and legal standards [10]. In other words, explainability plays a crucial role in making machine learning algorithms and AI not just transparent but also relatable and trustworthy in clinical settings.

Meanwhile, there has been an increasing worry about the possibility of machine learning models leading to biased decisions [11]. Examples include models displaying racial or gender biases in predicting patient outcomes [12], or algorithms that disproportionately favor certain demographics in resource allocation [13]. Such biased decision-making tools may result in unfair evaluations in clinical settings, ultimately harming patients who require care [14]. However, efforts to quantitatively evaluate fairness in prediction models for clinical practice are still scarce [15].

A model with high predictive accuracy does not guarantee the best clinical usage, as it might display unfavorable biases [16]. As a result, it is important to understand and quantify the trade-offs between accuracy and fairness in model selection.

Overall, the combined exploration and consideration of these aspects in a machine learning framework-based environment is not thoroughly investigated in this literature [17]. Therefore, to address these issues systematically, we propose a 3-stage machine learning framework on model assessment, selection, and explainability that integrates interpretability, fairness, as well as explainability in health care decision-making. Specifically, in the first stage, we develop and assess a range of models based on predictive accuracy and fairness. Next, we select the model that best balances accuracy and fairness using a novel trade-off curve. Finally, we explain the chosen model, aiming to provide deeper insights into its predictions for informed clinical decision-making. As a proof of concept, we apply our framework to predict cardiovascular disease (CVD) outcomes, myocardial infarction (MI), and stroke, among people with type 2 diabetes (T2D). With CVD being a leading cause of death in the United States, and patients with T2D being at elevated risk of CVD, it is urgent to develop accurate and fair predictive models that generate clinically reasonable predictions [16-19]. This study not only contributes to the advancement of AI in health care but also sets a precedent for future research in incorporating interpretability, fairness, and explainability into the machine learning model development framework, paving the way for more ethical, trustworthy, and effective solutions in medical informatics.

## Methods

### Overview

In this section, we first detail our study data and model development approach. Then, we describe our responsible framework for model assessment, selection, and explanation. This study is based on a secondary analysis of individual-level participant data from the Action to Control Cardiovascular Risk in Diabetes study (ACCORD 2001‐09, NCT00000620) by the National Heart, Lung, and Blood Institute (NHLBI) of United States [15]. Our code repository is included in Multimedia Appendix 1.

### Data

ACCORD (Action to Control Cardiovascular Risk in Diabetes) was a randomized, multicenter, double 2×2 factorial design study conducted at 77 clinical sites in North America. Participants were aged between 40 and 79 years, had T2D with a hemoglobin A1c (HbA_1c_) ≥ 7.5% (57 mmol/mol), and had previous evidence of CVD or cardiovascular risk factors. (eg, dyslipidemia, hypertension, smoking, or obesity). The primary outcome of ACCORD was determined based on the first instance of a significant CVD event, which was characterized by a combination of nonfatal MI, nonfatal stroke, or cardiovascular death. We extracted demographic, clinical, and biomarker data collected at baseline (study entry) from individual participants across trials for model development.

### Data Preprocessing

We focused on 2 primary CVD events, MI and stroke, as our study outcomes, and used patients’ demographic data, clinical risk factors, medication history, and pertinent biomarkers as predictors. All predictor variables were collected at the time of study enrollment, ensuring that our models use information available at the point of care. We prepared the study outcomes as binary variables: patients either experienced MI or stroke within the 5-year period, or they did not. In other words, the interpretable machine learning models act as classification tools to identify if a patient is at risk of experiencing these CVD events in the next 5 years.

**Figure 1.**
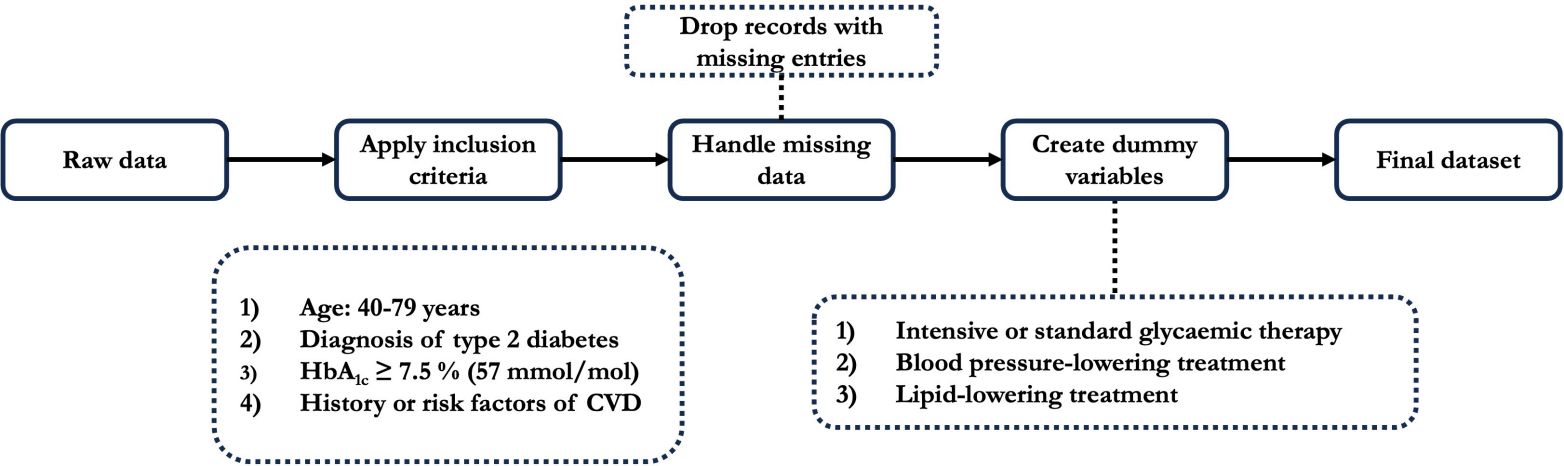
Data-preprocessing steps before model development.

We sourced candidate predictor variables for fatal or nonfatal MI and fatal or nonfatal stroke outcomes from eligibility screening or clinical examination data in ACCORD after applying the inclusion criteria. These predictors encompass demographic characteristics, clinical factors, medication history, and relevant biomarkers. Complete case models were constructed using all predictor variables, without using imputation, as only 616 out of 10,251 observations (6%) in the dataset had missing values across any of the predictors. We dropped the records with missing data and applied one-hot encoding for categorical predictors to obtain the final dataset for model development [20]. Specifically, creating dummy variables involved transforming categorical variables, such as treatment type (eg, intensive vs standard glycemic therapy) and medication history (eg, blood pressure- or lipid-lowering treatments), into binary indicators via one-hot encoding [21]. This step ensured that the categorical data was appropriately formatted for model development. This data-preprocessing pipeline is outlined in Figure 1.

### Machine Learning Model Development

We developed several machine learning models to demonstrate our proposed framework for fairness-aware model assessment and selection. Our framework is specifically designed for scenarios where model development, selection, and deployment are treated as separate processes. The machine learning models we consider include interpretable models (eg, linear and tree-based), semi-interpretable models, (eg, random forest), and common statistical and machine learning models, (eg, naïve Bayes), for binary classification of MI and stroke. In the following paragraphs, we provide details of the models developed in this research and describe the model-tuning procedure.

We evaluated a range of machine learning models, including linear models (GLMnet and OFS), tree-based models (CART and OCT), ensemble models (random forest and XGBoost), and other traditional machine learning approaches (naïve Bayes and SVM). Linear and tree-based models are generally considered interpretable due to their structure and parameterization [22,23], while ensemble models and other methods can achieve strong predictive performance but may be less directly interpretable. OFS formulates the logistic regression with L2 penalties into a binary convex optimization problem and solves it to optimality. Within the OFS framework, there are 2 key parameters: the regularization parameter (balancing model complexity against accuracy) and the sparsity parameter (enhancing interpretability by controlling feature count) [24]. OCT derives the tree by optimizing the tree structure (size) and decision rules simultaneously via mixed integer optimization [25]. The main hyperparameters in OCT include the maximum depth of a tree, the minimum leaf size, and the complexity parameter, playing a crucial role in preventing overfitting, ensuring stability, and fostering interpretability. We limited the max depth of an OCT to 3 and 4 to enhance interpretability by simplifying the decision structure. We remark that OCT exactly recovers the optimal tree (given fixed hyperparameters) at the cost of additional computational complexity, whereas CART uses heuristic splitting rules in branching nodes to generate a decision tree quickly. Detailed descriptions of each model’s structure, key hyperparameters, and training procedure are provided in Multimedia Appendix 2.

To build our models, we randomly divided our data using a 70‐30 train-test split, using 6745 out of 9635 for training and the remaining 2890 out of 9635 for testing. Then, with the training data, we applied 10-fold cross-validation for hyperparameter tuning. We also found that our data were class-imbalanced, and there were very few occurrences of CVD events. To address this class imbalance, we adjusted the weight assigned to each label during our hyperparameter tuning. Each model’s performance was estimated using both cross-validation on the training set and out-of-sample validation on the testing set.

### Responsible Framework for Model Assessment, Selection, and Explanation

**Figure 2.**
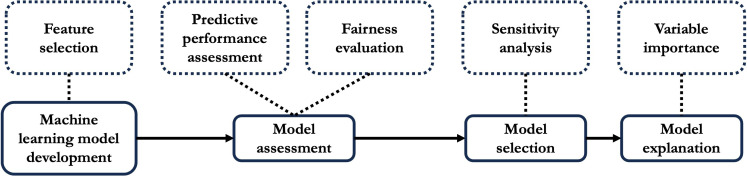
The proposed responsible framework for model assessment, selection, and explanation.

After building the machine learning models, it is desired to select a suitable model and investigate the relationship between predictors and the outcomes. In this section, we detail our responsible framework for model assessment, selection, and explanation. We outline this framework in Figure 2. Our proposed responsible framework consists of three main modules after model development: (1) model assessment, (2) model selection, and (3) model explanation. In the model assessment module, we assess each model’s performance in their predictive capabilities and fairness. Then, a sensitivity analysis of the trade-off between these performance metrics is carried out to aid model selection. Finally, for model explanation, we use a unified approach that combines multiple methods to explain the best-performing models. We developed our proposed responsible framework with R language (v4.3.1, R Foundation) and the following R libraries: Interpretable AI (v3.2.1, Interpretable AI), survival (v3.5.5, Mayo Clinic), GLMnet (v4.1.8, Stanford University), rpart (v4.1.19, Mayo Clinic), naivebayes (v1.0.0), and kernlab (v0.9.32, TU Wien).

#### Model Assessment

In this module, we describe the model assessment procedure for the developed interpretable models according to their predictive performance and fairness. To evaluate predictive accuracy, we considered four metrics: (1) area under the receiver operating characteristic curve (AUC), (2) sensitivity, (3) specificity, and (4) accuracy. AUC evaluates a model’s ability to distinguish between positive and negative classes, with higher values indicating better discriminative performance. It is particularly reliable for imbalanced data due to its threshold-independence. Sensitivity (true positive rate) measures the correct identification of actual positives (ie, patients developed MI or stroke), while specificity (true negative rate) assesses the correct identification of negatives. Accuracy quantifies the proportion of correct classification of the patients in the total cases examined. Sensitivity, specificity, and accuracy are threshold-dependent for models that output continuous scores (eg, predicted probabilities). Importantly, it is common to carefully select this classification threshold to optimize the model’s predictive performance, with respect to these performance metrics for specific applications. To address this challenge, we set a threshold which maximizes a weighted metric combining sensitivity and specificity, that is, $u\times sensitivity+\left(1-u\right)\times specificity$. Here, the weight parameter u is bounded between 0 and 1, and higher (resp., lower) values of u indicate a preference towards thresholds that emphasize sensitivity (resp., specificity). This approach has also been used in previous research [26] . In CVD management, both sensitivity and specificity play important roles in correctly identifying patients who are at risk of MI or stroke, along with those who are at low risk of these outcomes. Because correctly identifying those at high risk of MI or stroke is critical to initiating clinical interventions, we specifically use a value of $u=\frac{2}{3}$ in our analysis – leaning slightly toward higher sensitivity over specificity. This approach ensures that we assess a balanced performance in identifying both patients at risk and with low risk while accounting for clinical priorities.

To evaluate the predictive fairness of the machine learning models, we consider a fairness metric, the relative performance parity score (RPPS)*,* which is calculated by


$$RPPS:=1-max\underset{s}{\;}(|\frac{AUC_{s}-AUC}{AUC}|)$$

where s is a subpopulation in a protected attribute, eg, female in gender, and $AUC_{s}$ represents the conditional AUC conditioned on this subpopulation s. Notably, RPPS can be small when any subpopulation has disproportionally high or low AUC, compared with the overall AUC. On the other hand, if all subpopulations have AUC performance close to the overall AUC, then the RPPS will be large. We chose AUC as the primary metric because it is threshold-agnostic, providing a more comprehensive measure of model performance across different decision thresholds. Since this metric is a relative measure specific to each model’s output, it is suitable for a fair comparison across models, which befits our purpose in the model assessment module of the proposed responsible model selection framework. Moreover, the RPPS is flexible and can accommodate other commonly used performance metrics, such as accuracy or sensitivity, depending on the specific goals of the fairness evaluation.

#### Model Selection

After evaluating the models, selection could be based on either predictive performance or fairness. Ideally, one would choose a model that excels in both dimensions. However, this selection process becomes challenging when no such model exists within the considered model options. To address this, we propose a sensitivity analysis-based approach to enhance model selection. Specifically, we evaluated the weighted sum of accuracy *and RPPS*, with the weight ranging from 0 to 1, that is, w(Accuracy)+(1−w)RPPS,

where w is the prespecified weight between 0 and 1. Notably, when the weight is 1, this weighted sum simplifies to accuracy; conversely, when the weight is 0, it becomes the RPPS. This approach enables model selection according to the trade-off between predictive performance and fairness.

#### Model Explanation

To investigate the relationship between the predictors and outcomes, we developed a synergistic model explanation approach that combines models’ permutation variable importance, the SHAP method, and partial dependence plots. Notably, this explanation module does not establish clear causal relationships between features and adverse health outcomes; however, it helps clarify how the ML algorithms function for decision-making. Permutation variable importance assesses model explainability through feature significance [27]. That is, it measures the impact of each feature on a model’s predictive performance by shuffling the values of a feature while keeping others constant. We can then determine that feature’s importance based on the resulting performance decline, as measured by AUC. To enhance the reliability of our estimates, we bootstrapped 100 iterations: in each, we sampled the training data, trained a model, computed a baseline AUC, and determined permutation importance scores. This yielded multiple score sets for each bootstrapped sample. We then averaged the feature importance and provided 95% CIs. Features were ranked by mean importance and variability. A high mean importance means the model heavily depends on that feature, whereas high variability indicates inconsistent significance. Therefore, features with high variability warrant further examination, while those with high importance and low variability are consistently crucial.

Another component in our model explanation is the SHAP method. Essentially, SHAP assigns an importance measure, known as the Shapley value, to each feature. This Shapley value is calculated by averaging the differences in the model’s predictions with and without the feature across all possible subsets of features, which can be viewed as the expected effects of the feature on the prediction. Importantly, the sum of all Shapley values equals the prediction value to ensure consistency across all features. The Shapley value effectively captures the average marginal contribution of each feature, providing a comprehensive explanation of the model’s behavior. For interpretability, we proposed to use the relative Shapley value (the marginal contribution of each feature relative to the prediction value) in our analysis.

To further visualize and understand the relationship between features and predicted outcomes, we also consider Partial Dependence plots as another explanation method. Partial Dependence plots are widely used and show how changing a feature value affects model outputs, by fixing all other features [28]. This explanation method is chosen over the SHAP’s built-in dependence plot function because Partial Dependence plots tend to be more intuitive in clinical settings [29]. Specifically, when creating a partial dependence plot, we replace the value of a feature with values in its range to compute average model outputs on this feature’s range across data samples. In our analysis, in addition to risk predictions, we used log odds as model outcomes to provide a better interpretation of features and outcomes. In essence, a positive log-odds value indicates a higher likelihood of the event occurring, a negative value indicates a lower likelihood, and a value of zero represents a 50% probability. We made the necessary adjustments for categorical and continuous features and included data distribution information to bolster the reliability of our analysis. This approach enables us to pinpoint areas where the model’s output is robustly supported by data, as well as identify regions where predictions may be less dependable due to data scarcity.

We stress that both permutation variable importance and the SHAP method can provide broader insights into the overall effects of specific features on model outputs. Partial dependence plots, on the other hand, provide a detailed visualization to uncover the direct relationship between each feature and the predicted outcome. By integrating permutation variable importance, the SHAP method, and partial dependence plots, we deliver a holistic model explanation for users. This synergy enhances interpretability and trust in the model’s predictions, making the analysis more actionable in clinical settings.

### Ethical Consideration

This study was approved by the Institutional Review Board at the Georgia Institute of Technology under Protocol No. H22333. All participants in the ACCORD trial provided written informed consent. The ACCORD contained de-identified data only. Participants in the ACCORD trial were not paid for their participation.

## Results

### Study Sample

Our study data included 9635 participants, with 616 (6% of the total 10,251) excluded due to missing data on predictor variables (Table 1). The mean (SD) age was 62.8 (6.7) years. Women made up 3,662 (38%) of the sample. The racial and ethnic make-up of our study data included 1834 (19%) non-Hispanic Black participants, 678 (7%) Hispanic or Latino participants, and 7123 (74%) non-Hispanic White participants. In addition, 3437 (36%) of participants had a history of CVD. The mean (SD) body mass index was 32.2 (5.4) kg/m^2^, systolic blood pressure was 136.5 (17.1) mm Hg, and diastolic blood pressure was 74.9 (10.7) mm Hg. Of the sample, 880 (9.1%) experienced an MI, and 197 (2%) had a stroke during the follow-up period.

**Table 1.** Summary of study sample characteristics.

Variable	Value (n=9635)
Demographics	
Age, years, mean (SD)	62.8 (6.66)
Aged 75 years or older, n (%)	521 (5.4)
Gender, n (%)	
Women	3662 (38)
Men	5973 (62)
Race and ethnicity, n (%)	
Non-Hispanic Black	1834 (19)
Hispanic or Latino	678 (7)
Non-Hispanic White	7123 (74)
Tobacco usage, current, n (%)	1179 (12)
BMI, kg/m^2^, mean (SD)	32.2 (5.4)
Blood pressure, mean (SD)	
Systolic, mm Hg	136.5 (17.1)
Diastolic, mm Hg	74.9 (10.7)
Heart rate, bpm, mean (SD)	72.7 (11.8)
History of CVD^a^, n (%)	3437 (36)
Drug usage, n (%)	
Blood pressure-lowering drugs	8109 (94)
Oral diabetes drugs (including metformin)	8024 (83)
Insulin treatment	3403 (35)
Statins	6148 (64)
Fibrates	601 (6)
Anticoagulant use	303 (3)
Nonsteroidal anti-inflammatory use	851 (9)
Platelet aggregate inhibitor use	466 (5)
Daily aspirin use	5274 (55)
Biomarkers, mean (SD)	
HbA_1c_^b^, %	8.3 (1.1)
HbA_1c_, mmol/mol	67 (9)
Total cholesterol, mg/dL	183.2 (41.7)
HDL cholesterol, mg/dL	41.8 (11.6)
LDL cholesterol, mg/dL	104.7 (33.8)
Triglycerides, mg/dL	190.7 (145.8)
Fasting plasma glucose, mg/dL	175.3 (55.8)
Alanine aminotransferase, IU/L	27.5 (16.0)
Creatine phosphokinase, IU/L	140.3 (130.2)
Serum potassium, mmol/L	4.5 (0.5)
Serum creatinine, mg/dL	0.9 (0.2)
Estimated glomerular filtration rate, mL/min/1.73 m^2^	90.9 (27.3)
Urine albumin, mg/dL	10.7 (37.3)
Urine creatinine, mg/dL	127.3 (65.4)
Urine albumin: creatinine ratio, mg/g	99.2 (359.4)

a CVD: cardiovascular disease.

b HbA_1c_: Hemoglobin A1c.

### Predictive Performance

The predictive accuracy of our machine learning models is summarized in Table 2.

**Table 2.** Summary of predictive performance for machine learning models predicting risk of myocardial infarction and stroke, optimizing thresholds for $\frac{2}{3}\times Sensitivity+\frac{1}{3}\times Specificity$.

Machine learning model	AUC	Sensitivity	Specificity	Accuracy
MI^a^				
OCT	0.687	0.782^b^	0.554	0.565
Naïve Bayes	0.694	0.563	0.768^b^	0.420
Random forest	0.716^b^	0.746	0.640	0.645
SVM	0.581	0.711	0.436	0.450
XGBoost	0.695	0.782	0.554	0.565
GLMnet	0.704	0.629	0.666	0.664^b^
OFS	0.705	0.671	0.642	0.644
Stroke				
OCT	0.625	0.771	0.546	0.550
Naïve Bayes	0.703	0.694	0.708	0.708
Random forest	0.716	0.510	0.839^b^	0.833^b^
SVM	0.624	0.816^b^	0.426	0.568
XGBoost	0.714	0.653	0.735	0.734
GLMnet	0.700	0.625	0.715	0.714
OFS	0.731^b^	0.646	0.716	0.715

a MI: myocardial infarction.

b The best-performing model for each performance metric.

For classification of MI, the OCT model achieved the highest sensitivity at 0.782 with a specificity of 0.554 and AUC of 0.687. Random forest achieved the highest AUC at 0.716, with a sensitivity of 0.746, moderate specificity (0.640), and accuracy (0.645). XGBoost shows similar characteristics, with an AUC of 0.695, sensitivity matching OCT at 0.782, but lower specificity (0.554) and accuracy (0.565). GLMnet, despite its lower sensitivity (0.629), has a high AUC of 0.704 and the best specificity (0.666) and accuracy (0.664) among the models. Naïve Bayes, while exhibiting lower sensitivity (0.563), has the highest specificity (0.768), though an accuracy of 0.420.

For stroke classification, our results indicate that naïve Bayes provides the most balanced results across AUC (0.703), sensitivity (0.694), specificity (0.708), and accuracy (0.708). On the other hand, the Random forest model, despite having a lower sensitivity, achieves a high AUC (0.716) and the highest specificity (0.839), along with an accuracy of 0.833 – indicating its strong performance in correctly identifying non-stroke cases at the expense of missing some stroke cases. Similarly, XGBoost performs exceptionally well on AUC (0.714), accuracy (0.734), and specificity (0.735), with a low sensitivity (0.653). In contrast, the OCT model has the second-lowest AUC (0.625) and accuracy (0.550), indicating it may be the least effective model for stroke prediction. This lower performance suggests that OCT might not be suitable for accurate stroke prediction compared with the other models considered.

### Fairness

In Table 3, we present AUC stratified by gender and race across all of the developed interpretable machine learning models for MI and stroke classification, along with the RPPS scores for gender and race. The conditional AUC on each subgroup is calculated by classification results in the test set.

**Table 3.** Area under the curve and Relative Parity of Performance Scores of myocardial infarction and stroke for gender and race across all machine learning models.

Machine learning model	AUC^a^		RPPS^b^	AUC		RPPS
	Men	Women	Gender	Black	White	Race
MI^c^						
Naïve Bayes	0.667	0.708	0.961	0.745	0.681	0.927
SVM	0.558	0.675	0.838	0.605	0.591	0.959
Random forest	0.673	0.760	0.939	0.770	0.704	0.925
XGBoost	0.655	0.753	0.917	0.720	0.690	0.964
GLMnet	0.689	0.702	0.979^d^	0.681	0.706	0.967^d^
OCT	0.642	0.763	0.889	0.710	0.682	0.967^d^
OFS	0.688	0.716	0.976	0.668	0.708	0.948
Stroke						
Naïve Bayes	0.679	0.662	0.942	0.733	0.629	0.895
SVM	0.622	0.615	0.986^d^	0.600	0.609	0.962
Random forest	0.783	0.673	0.906	0.741	0.696	0.965
XGBoost	0.773	0.645	0.903	0.718	0.702	0.983^d^
GLMnet	0.714	0.727	0.961	0.685	0.698	0.979
OCT	0.595	0.670	0.928	0.701	0.582	0.878
OFS	0.687	0.787	0.923	0.692	0.743	0.947

a AUC: area under the curve.

b RPPS: Relative Parity of Performance Scores.

c MI: myocardial infarction.

d The best-performing model for each performance metric.

For MI classification, besides the SVM, which has the lowest RPPS (0.838), the random forest model shows a substantial disparity in AUC between men (0.673) and women (0.760), resulting in a lower RPPS (0.939), which indicates higher gender disparities. Similarly, XGBoost exhibits a high AUC for women (0.753) compared with men (0.655), leading to an RPPS of 0.917, further highlighting the model’s performance gaps across genders. On the other hand, the GLMnet and OCT model demonstrate the highest RPPS (0.979), suggesting minimal performance disparities between Black (0.681) and White (0.706) subgroups for GLMnet, and Black (0.710) and White (0.682) subgroups for OCT. In contrast, the random forest model shows greater differences in performance by race with an AUC of 0.770 for non-Hispanic Black people and 0.704 for non-Hispanic White people, resulting in a lower RPPS (0.925). Importantly, for most models, the RPPS scores indicate fairer results among race groups, compared with gender, for predicting MI events.

For stroke classification, the XGBoost model exhibits a significant difference in AUC between men (0.773) and women (0.645), resulting in a lower RPPS (0.903), highlighting pronounced differences in predictive accuracy by gender. Similarly, the random forest model, with a high AUC for men (0.783) compared with women (0.673), leads to an RPPS of 0.906. Conversely, the XGBoost model shows a relatively high RPPS (0.983), implying minimal performance differences between non-Hispanic Black (0.718) and non-Hispanic White (0.702) people. However, the naïve Bayes model demonstrates greater differences by race with an AUC of 0.733 for non-Hispanic Black people and 0.629 for non-Hispanic White people, resulting in a lower RPPS (0.895). Importantly, in contrast to our findings in MI classification tasks, the RPPS scores reveal better performance parity among gender groups compared with racial groups in classifying stroke events.

We also analyzed the coefficients of the one-hot encoded gender and race variable for GLMnet. For the variable “female=1”, coefficient values were -0.324 in the model for MI and -0.250 in the model for stroke, suggesting that women are associated with a lower predicted risk of MI and stroke compared with men. For race, the variable “black=1” had a coefficient value of -0.192 in the model for MI, which indicates a lower predicted risk of MI for non-Hispanic Black people compared with non-Hispanic White people. For stroke, the model does not provide a coefficient for this predictor, indicating it was not significant in this context. These findings highlight the potential disparities in model predictions based on demographic factors, emphasizing the need to consider these variables when developing and evaluating predictive models in CVD classification.

### Model Selection

**Figure 3.**
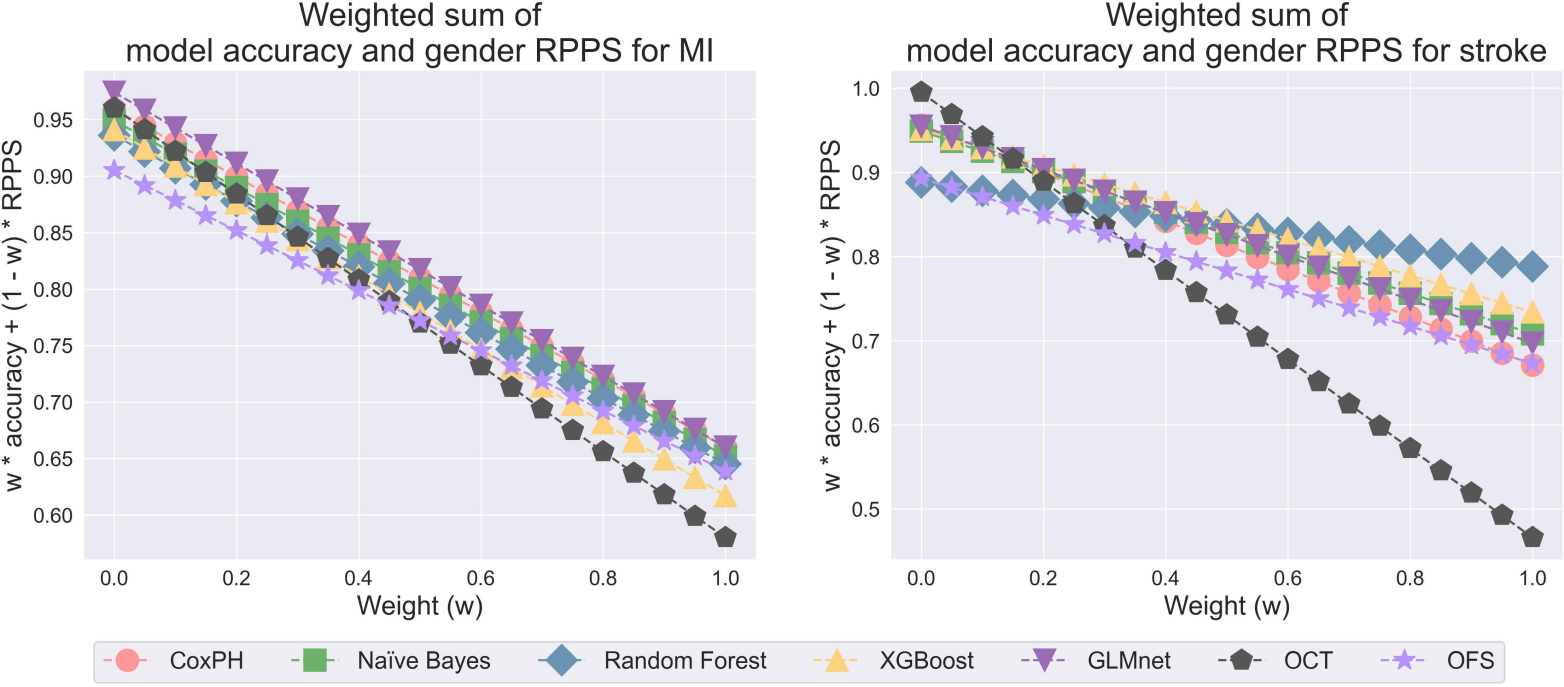
Weighted sum of model accuracy and gender Relative Parity of Performance Scores for myocardial infarction and stroke. MI: myocardial infarction; RPPS: Relative Parity of Performance Scores.

We applied our model selection approach to identify the most preferable predictive models for MI and stroke classification. From our analyses, we have found that these models can exhibit higher disparities across gender subgroups, compared with race. Consequently, alongside overall predictive performance (overall accuracy), we incorporated gender RPPS scores into our model selection criteria. The weighted sum of accuracy and RPPS for MI and stroke classification among all machine learning models is presented in Figure 3. Recall that, when the weight is selected to be 0, this weighted sum represents RPPS; and when the weight is 1, it represents accuracy. For MI classification, the GLMnet model consistently demonstrated the highest values across most weights, indicating its superior balance of accuracy and fairness (Gender RPPS). This is particularly evident as the weight approaches 0, with a gender RPPS of 0.974. On the other hand, for stroke classification, the best-performing model varies across different weights. At lower weights (0 to 0.2), the OCT model showed the highest values due to its strong Gender RPPS. As the weight increases, the GLMnet model starts to dominate from the weight is 0.25 to 0.6, maintaining a good balance between accuracy and Gender RPPS. Moving to higher weights (0.65 onwards), the random forest model outperformed others, with its accuracy dominating the performance at around 0.788. These results indicated that, for stroke classification, when users prioritize model fairness, the OCT model is the best. For users who are neutral regarding the tradeoff between fairness and accuracy, GLMnet emerges as a suitable option. Lastly, random forest is recommended for users with a strong preference for model accuracy. Accordingly, we assumed users want to balance accuracy and fairness and proceed to analyze the relationship between variables and outcomes using GLMnet for both MI and stroke classification.

### Model Explanation

**Figure 4.**
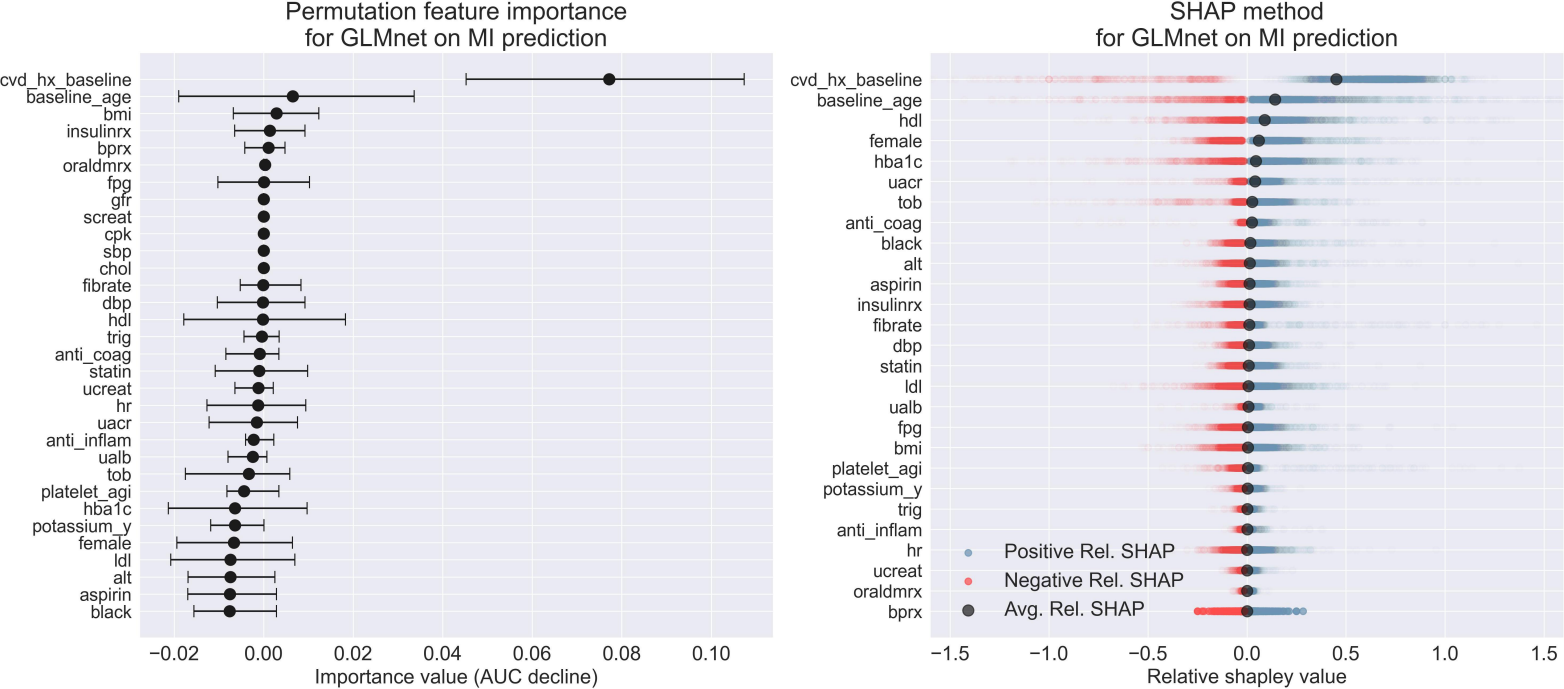
Permutation variable importance and Shapley Additive Explanations method for GLMnet on myocardial infarction classification. AUC: area under the curve; MI: myocardial infarction; SHAP: Shapley Additive Explanations.

We analyzed the relationship between features and outcomes for predicting MI and stroke using GLMnet, with our synergistic model explanation approach that integrates the permutation variable importance, the SHAP method, and the partial dependence plots. Figures4  5 display the permutation variable importance measures, along with their 95% CIs, and the SHAP method for the GLMnet model in predicting MI and stroke, respectively. In addition, partial dependence plots for GLMnet for MI and stroke are shown in Figures6  7, respectively.

**Figure 5.**
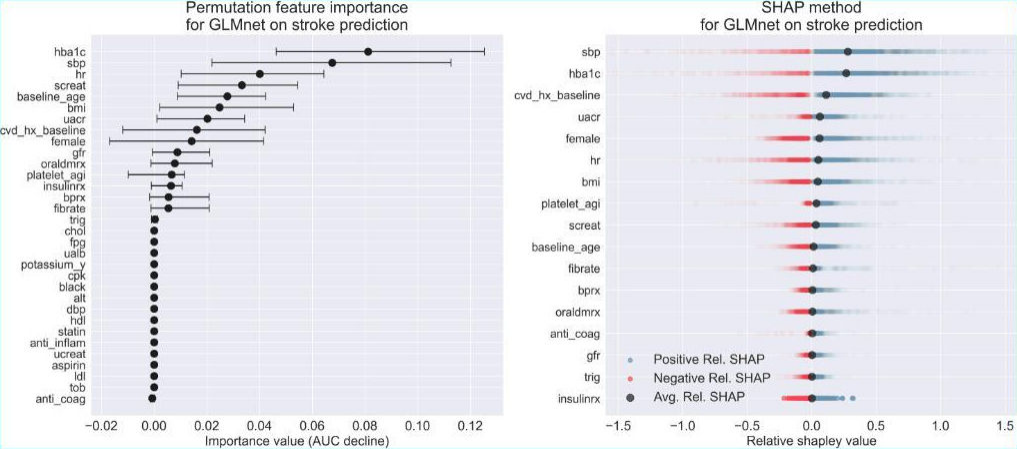
Permutation variable importance and Shapley Additive Explanations method for GLMnet on stroke classification. CVD: cardiovascular disease; HbA_1c_: hemoglobin A1c.

**Figure 6.**
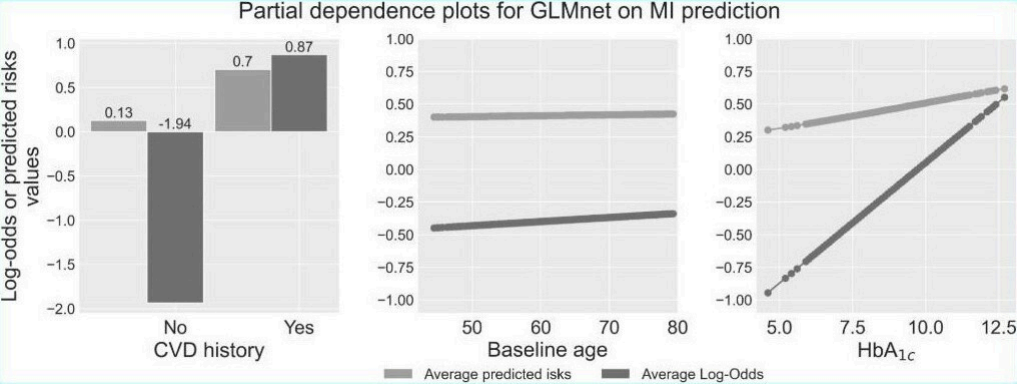
Partial dependence plots for GLMnet on myocardial infarction classification. AUC: area under the curve; SHAP: Shapley Additive Explanations.

**Figure 7.**
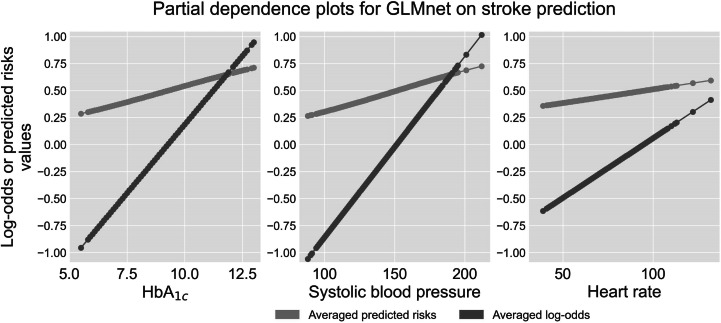
Partial dependence plots for GLMnet on stroke classification.

From Figure 4, we observe that the permutation variable importance technique highlights the history of CVD (cvd_hx_baseline) as the most critical predictor for GLMnet to accurately predicting MI risks. Notably, its mean importance value (0.078) is nearly twelve times greater than that of the second most important variable, age (baseline_age), which has a mean importance value of 0.006. Moreover, while the 95% CIs of the permutation importance for CVD history is (0.045, 0.107), which is among the widest in the feature space, its lower confidence interval is still higher than the upper confidence interval of any other features. These results imply the significant role of people’s CVD history to make accurate classification using GLMnet. In addition, BMI, and insulin treatment (insulinrx) also rank highly in this importance metric, with mean importance values of 0.002 and 0.0013, respectively. Some variables show negative values in permutation importance, which could be related to overfitting. Since the negative values are small in magnitude in our case, it could be that the model has relied too heavily on noise rather than true signal for those predictors. Now, from the SHAP method, we observed that the history of CVD and age emerge as the 2 most influential features in driving GLMnet’s MI classification, as indicated by their average relative Shapley values of 0.45 and 0.14, respectively. This suggests that these 2 features have a general tendency to positively contribute to the prediction value. However, the individual relative Shapley values for the features can widely range from negative to positive, reflecting the varying marginal contributions of the features across individuals. For example, it appears that the distribution of the relative Shapley values for CVD history is a mixture of 2 highly separated distributions, which again implies that the GLMnet is substantially sensitive to CVD history. Furthermore, our findings indicate that although insulin treatment is highlighted as one of the top features in permutation variable importance, its mean relative Shapley value is relatively low compared with other features. Conversely, HbA_1c_ ranks highly using the SHAP method. Since both insulin treatment and HbA_1c_ are indicative of an individual’s diabetic status, this suggests that accurately predicting MI also heavily depends on the diabetes status of the individual. Consequently, we next analyze the partial dependence plots for CVD history, age, and HbA_1c_ to draw actionable insights.

The partial dependence plots in Figure 6 illustrate that individuals with a history of CVD can have log odds of 0.87 (risk of 0.7), while those without CVD can have log odds as low as −1.94 (risk of 0.13). In addition, the plots show that 44-year-old individuals in the test set have log-odds of −0.44 (risk of 0.4) for developing MI. As age increases to 79 years, the risk gradually rises to log-odds of −0.33 (risk of 0.42). Furthermore, the plots reveal that HbA_1c_ levels significantly impact risk: with HbA_1c_ as low as 4.6%, the log-odds are −0.94 (risk of 0.3), but as HbA_1c_ rises to 12.7%, the log-odds increase drastically to 0.55 (risk of 0.61). These results highlight the importance of considering both CVD history and key biomarkers such as HbA_1c_ in assessing MI risk. Notably, although partial dependence plots do not necessarily reveal causation between features and risks, the steep increase in risk associated with higher HbA_1c_ levels underscores the critical role of diabetes management in preventing MI.

Next, we conduct a model explanation analysis for GLMnet in predicting stroke. As shown in Figure 7, HbA_1c_, systolic blood pressure (SBP), and heart rate (HR) have the highest mean permutation importance values (0.08, 0.06, and 0.01, respectively). Other significant features include serum creatinine (screat), age, and BMI. These features exhibit wide confidence intervals for their permutation importance values, suggesting that their influence on the model can vary depending on the patient cohort. Moreover, HbA_1c_ and SBP consistently show the highest positive marginal contributions to risk predictions, with average relative Shapley values of 0.28 and 0.26, respectively. Heart rate, with a relative Shapley value of 0.05, is also a significant predictor for stroke using GLMnet. Although there are instances where these features negatively contribute to risk prediction, their relative Shapley values are predominantly positive. This distribution indicates a general tendency for these features to positively drive the predicted values, highlighting their crucial role in the model’s stroke risk predictions. Since these features are essential for accurately assessing stroke risk, we now derive actionable insights from the partial dependence plots (Figure 7).

The partial dependence plots in Figure 7 provide visualizations of how HbA_1c_, systolic blood pressure, and heart rate influence stroke classification using the GLMnet model. We noticed that all 3 features exhibit a clear positive relationship between them and the predicted risk of stroke. For HbA_1c_, as levels increase from 5.5% to 13%, there is a noticeable rise in both log-odds and predicted risk, moving from around −0.95 to 0.94 in log-odds and from 0.28 to 0.71 in predicted risk. For systolic blood pressure, the log-odds of developing stroke can reach 1.01 from −1.05 and from 0.26 to 0.725 in predicted risk as systolic blood pressure increases from 88 mm Hg to 212 mm Hg. Notably, the rate of increase in stroke risk with rising systolic blood pressure is less steep compared with the rate observed with increasing HbA_1c_ levels. Finally, the log-odds of developing stroke can reach 0.41 from -0.61 and from 0.35 to 0.59 in predicted risk as heart rate increases from 39 bpm to 132 bpm. Comparatively, the rate of increase in risk with heart rate is the most moderate among these features. This indicates that managing HbA_1c_ and systolic blood pressure could be more effective in preventing stroke. Overall, our model explanation analyses highlight the importance of managing blood glucose levels, blood pressure, and heart rate to mitigate the risk of stroke.

## Discussion

### Principal Findings and Comparison With Previous Works

In this study, we designed a responsible framework that evaluates various machine learning models by comparing these models’ predictive accuracy and fairness metrics, while also providing model explanation. We then applied this framework for classification of MI and stroke, demonstrating its effectiveness in highlighting the importance of in-depth analyses of interpretable machine learning models between these 3 dimensions.

Our results demonstrate that complex models are not necessarily always better than simple, interpretable models—especially for high-stakes decisions such as those encountered in medicine [30]. Importantly. while the investigation of predictive accuracy have been a focus of several previous studies in health care AI, the concurrent evaluation of fairness in machine learning models is inconsistent and lacking [12]. The fairness analysis of our predictive models reveals both strengths and areas of concern in terms of gender and racial bias. In MI prediction, while some models like the SVM and XGBoost demonstrate gender biases, others such as GLMnet and OFS models show more balanced performance across gender and racial groups. This indicates that the most accurate models are not necessarily the fairest, and vice versa. These trade-offs highlight the importance of carefully selecting and tuning models to balance accuracy and fairness in medical decision-making. For stroke prediction, while some models, that are, GLMnet and SVM, exhibit balanced performance for both gender and race, other models, that are, XGBoost, present bias for a gender. This finding highlights the complex nature of fairness in such models, where different types of biases may manifest depending on the outcome being predicted and the model used. These findings underscore the importance of continuously monitoring and evaluating models for fairness. Our study provides a comprehensive analysis of fairness across different models and conditions, paving the way for more equitable AI applications in health care.

While there are multiple important criteria for model selection, such as predictive performance and fairness, a unified approach to guide this selection process is lacking. To address this, our analysis presents a sensitivity analysis based selection procedure based on users’ preferences over the selection criteria. Our findings suggest that in some prediction tasks, users’ preferences have minimal effect on the best-performing model (eg, GLMnet for MI prediction). However, in other tasks, for example, stroke prediction, users' preferences can significantly influence the selected model. Our study highlights the necessity of considering user preferences in model selection to ensure optimal outcomes for different prediction tasks.

Finally, our study demonstrates the potential role that our integrated explanation method (ie, the combination of permutation variable importance, the SHAP method, and partial dependence plots) can play in enhancing clinicians’ understanding and trust of model-based predictions. For instance, the permutation variable importance measures, the Shapley values and partial dependence plots provide a clear visual representation of how key features like HbA_1c_ and systolic blood pressure influence the model’s risk prediction for MI and Stroke. Such visual explanations can provide actionable insights and be augmented with existing clinical knowledge to help validate the quality of model-generated risk estimates [31]. These visualizations can also help clinical experts explain the unknown complex relationships between various risk factors and adverse outcomes [32].

This study has some limitations. The proposed framework was tested solely on CVD classification using the ACCORD dataset. While it has shown promise in this context, its effectiveness in other disease areas needs further investigation. Moreover, we relied on baseline information collected at the start of the study for model development and did not fully account for how things can change over time in the real world. Looking ahead, we would like to make sure our models stay accurate and up to date as treatment strategies and clinical guidelines evolve. One way to do this is by applying our framework on more recent datasets or on data that captures changes over time. These steps could give us a better understanding of how changing clinical practices might affect prediction modeling.

### Conclusions

In this research, we proposed a 3-stage responsible framework for developing, selecting, and explaining machine learning models, emphasizing the trade-off between predictive accuracy and fairness in health care applications. By quantifying this trade-off using AUC and RPPS, we provided a structured approach to responsible model selection. After selecting the final model, we proposed an integrated explanation method to offer insights into the relationships between features and outcomes. Applying this framework to predict MI and stroke among people with T2D, we demonstrated its effectiveness and potential to improve the development and evaluation of machine learning models for clinical practice. We anticipate that our framework is generalizable and can be applied to other clinical prediction tasks, potentially increasing the trustworthiness and acceptance of machine learning models among clinicians and patients.

Our framework highlights the importance of combining interpretability, explainability, and fairness in building, selecting, and explaining machine learning models. This integration is crucial not only for enhancing model performance [33], but also for addressing ethical and legal considerations [34]. These principles both help verify model results against clinical literature [35], and fosters acceptance and trust among health care stakeholders [36,37]. By fully embracing these aspects, our framework paves the way for more responsible, ethical, and transparent AI applications in health care.

## Supplementary material

10.2196/66200 Multimedia Appendix 1 Code repository.

10.2196/66200 Multimedia Appendix 2 Details of model descriptions and hyperparameters, along with explanation methods.

## Abbreviations

ACCORD: Action to Control Cardiovascular Risk in Diabetes study
AI: Artificial Intelligence
AUC: area under the curve
CVD: cardiovascular disease
HbA_1c_: Hemoglobin A1c
MI: myocardial infarction
NHLBI: National Heart, Lung, and Blood Institute
RPPS: Relative Parity of Performance Scores
SHAP: Shapley Additive Explanations
T2D: type 2 diabetes
