# Supplementary material for: A Responsible Framework for Assessing, Selecting, and Explaining Machine Learning Models in Cardiovascular Disease Outcomes Among People With Type 2 Diabetes: Methodology and Validation Study
Source: JMIR Med Inform. 2025 Jun 27;13:e66200. doi: 10.2196/66200 (PMC12256707; doi:10.2196/66200)
Supplement: Multimedia Appendix 1 [file medinform-v13-e66200-s001.zip › Code/Readme.docx]

**README**

**A Responsible Framework for Assessing, Selecting, and Explaining Machine Learning Models in Cardiovascular Disease Outcomes Among People with Type 2 Diabetes: Methodology and Validation**

***MI_main_analysis.R***
This script begins by loading the ACCORD dataset (accord_sbasu_cut.RData) from [Sanjay Basu’s GitHub repository](https://github.com/sanjaybasu/t2dmriskeqns.git) and initializes the necessary R packages. It then creates a variety of indicator variables (for medication usage, race/ethnicity, and more) and calculates important baseline measures such as BMI. Myocardial infarction (MI) is defined as a binary outcome.

After preparing the data, we split it into training and testing sets and conducts grid searches to fine-tune model hyperparameters for the following machine-learning algorithms, including Random Forest, XGBoost, GLMnet, Optimal Classification Tree (OCT), Optimal Feature Selection (OFS), Naïve Bayes, and Support Vector Machine. Each model’s performance is evaluated in terms of overall metrics (AUC, sensitivity, specificity, and accuracy) and subgroup metrics, providing a thorough assessment of predictive performance and fairness.

***MI_Shapley_Permutation_Importance.R***
This script covers two interpretability techniques: SHAP values and Permutation Feature Importance.

1. **SHAP Value Computation**
   It calculates instance-level SHAP values to break down the model’s predictions and show the positive or negative contributions of each feature
2. **Permutation Feature Importance**
   In addition, it performs a baseline AUC computation, permutes each feature, and measures the corresponding decrease in predictive performance. This procedure ranks the features by how important they are to model accuracy and visualizes the results with confidence intervals.

***MI_partial.R***
This script focuses on partial dependence analysis of the best-performing model from the main analysis. It generates partial dependence plots (PDPs) for key features, illustrating how predicted probabilities and log-odds of MI vary with changes in individual predictors.

***Model_Selection.ipynb***
This Jupyter notebook provides a flexible framework for analyzing trade-offs between accuracy and fairness, as measured by the Relative Parity Probability Score (RPPS). By computing a weighted sum of these metrics for each model, it enables users to explore how varying the importance of accuracy versus fairness alters overall model ranking. Users can easily substitute other metrics of interest, underscoring the notebook’s adaptability.

In addition, the notebook refines the visualization of permutation importance, SHAP plots, and partial dependence plots, producing clear and user-friendly figures. Taken together, these features offer a comprehensive and highly intuitive view of model performance, making the notebook an invaluable resource for model selection.
