# Supplementary material for: A Responsible Framework for Assessing, Selecting, and Explaining Machine Learning Models in Cardiovascular Disease Outcomes Among People With Type 2 Diabetes: Methodology and Validation Study
Source: JMIR Med Inform. 2025 Jun 27;13:e66200. doi: 10.2196/66200 (PMC12256707; doi:10.2196/66200)
Supplement: Multimedia Appendix 2 [file medinform-v13-e66200-s002.docx]

**MULTIMEDIA APPENDIX 2**

**Model Descriptions and Hyperparameters**

We provide the full details of each model’s structure, key hyperparameters, and training procedures. Some models (OFS and OCT) have already been described extensively in the main text, but we include their original descriptions here for completeness. All references correspond to those listed in the main manuscript’s reference section.

**Linear Models (GLMnet and OFS)**
Linear models are generally considered to be interpretable due to their linear combination of inputs, making it easier to understand the effect of each feature on the predicted outcome [18]. We tested two types of linear models: GLMnet and Optimal Feature Selection (OFS). GLMnet, the Lasso and Elastic-Net Regularized Generalized Linear Model, is an algorithm for generalized linear models that uses both L1 (LASSO) and L2 (ridge) regularization to reduce overfitting [19]. Since our outcome is binary, we used a logistic link function in our implementation. The key hyperparameters in GLMnet are alpha (balancing L1 and L2 regularization) and lambda (penalty strength). The elastic net (beta) penalty, which is a convex combination of L1 and L2 penalties, offers the benefits of both LASSO and ridge regression.

On the other hand, OFS formulates the logistic regression with L2 penalties into a binary convex optimization problem and solves it to optimality. Within the OFS framework, there are two key parameters: the regularization parameter (balancing model complexity against accuracy) and the sparsity parameter (enhancing interpretability by controlling feature count) [20]. Both GLMnet and OFS aim to mitigate overfitting by inducing sparsity in the model [20–21].

**Tree-based Models (CART and OCT)**
Tree-based models are generally considered to be interpretable due to their structure [23]. We designed and assessed two types of tree-based models: the Classification and Regression Tree (CART) and Optimal Classification Tree (OCT). CART recursively partitions the features to generate “pure” leaf nodes that can accurately identify the outcome variable, forming a tree-like structure [24]. The main hyperparameters in CART include the maximum depth of a tree, the minimum leaf size, splitting criterion, etc. Each split in the tree is derived from solving an optimization problem. CART’s primary drawback is its greedy approach since it determines splits in isolation, neglecting the impact on subsequent splits, which can result in suboptimal tree structure.

In contrast, OCT derives the tree by optimizing the tree structure (size) and decision rules simultaneously via mixed integer optimization [25]. The main hyperparameters in OCT include the maximum depth of a tree, the minimum leaf size, and the complexity parameter, playing a crucial role in preventing overfitting, ensuring stability, and fostering interpretability. We limited the max depth of an OCT to 3 and 4 to enhance interpretability by simplifying the decision structure. We remark that OCT exactly recovers the optimal tree (given fixed hyperparameters) at the cost of additional computational complexity, whereas CART uses heuristic splitting rules in branching nodes to generate a decision tree quickly.

**Ensemble Models (Random Forest and XGBoost)**
For semi-interpretable models, we considered two ensemble models: Random Forest and XGBoost. Random Forest uses bootstrapping to create multiple trees (CART models), aggregating predictions through majority voting [26]. Key hyperparameters include node size, the number of trees, and features sampled for splitting.

XGBoost constructs trees sequentially, addressing errors from the previous tree. Hyperparameters include learning rate, tree depth, subsample ratio, and regularization term [27]. For both models, we limited the number of splits to 5 for future comparisons with the Optimal Classification Tree. While not inherently interpretable, Random Forest and XGBoost are recognized for predictive performance, serving as high-quality benchmarks in our study.

**Other Statistical/Machine Learning Models (Naïve Bayes and SVM)**
Other statistical learning and machine learning models include Naïve Bayes and Support Vector Machine (SVM) [28]. Naïve Bayes is a probabilistic classifier based on Bayes’ Theorem. In the binary classification problem, Naïve Bayes computes the probability of each outcome, given a set of features, and assigns the outcome with the highest probability. For Naïve Bayes, we tuned the smoothing parameter to stabilize the calculation of conditional probabilities and the bandwidth parameter of kernel density to avoid overfitting.

SVM is a supervised machine learning model designed for classification tasks. The key parameters for SVM include cost and gamma. SVM aims to search for a decision boundary that can divide the positive and negative outcomes. We tuned the margin of this decision boundary for SVM to balance between overfitting and misclassification [22].

**Explanation Methods (Partial Dependence Plots and SHAP’s Built-in Dependence Plots)**

In our analysis, we used Partial Dependence plots instead of SHAP’s built-in dependence plot function because Partial Dependence plots are more straightforward and easier to interpret in clinical settings. Partial Dependence plots visualize how changes in one feature affect the model’s predicted outcomes by averaging the output over all other features. This approach allows us to isolate that feature’s direct effect, making it easier to understand.

SHAP dependence plots, on the other hand, show how a feature’s value influences its Shapley value, representing the feature’s contribution to the model’s prediction. While SHAP dependence plots can highlight feature interactions by coloring points based on another feature, this added complexity makes them harder to interpret.

In our case, we prioritize displaying the direct impact of a single feature on predicted outcomes, as it is more intuitive and easier to comprehend in clinical contexts.

**R Scripts and Interactive Jupyter Notebook**

We provided our sample R scripts for model assessment and explanation. A Jupyter notebook is also provided for model selection and sensitivity analysis.
